# Piezoelectric sensor based on graphene-doped PVDF nanofibers for sign language translation

**DOI:** 10.3762/bjnano.11.148

**Published:** 2020-11-02

**Authors:** Shuai Yang, Xiaojing Cui, Rui Guo, Zhiyi Zhang, Shengbo Sang, Hulin Zhang

**Affiliations:** 1 Micro Nano System Research Center, College of Information and Computer, Taiyuan University of Technology, Taiyuan, 030024, China; 2 College of Textile Engineering, Taiyuan University of Technology, Taiyuan, 030024, China

**Keywords:** motion sensor, piezoelectric, polyvinylidene fluoride (PVDF), self-powered, sign language translation

## Abstract

The tracking of body motion, such as bending or twisting, plays an important role in modern sign language translation. Here, a subtle flexible self-powered piezoelectric sensor (PES) made of graphene (GR)-doped polyvinylidene fluoride (PVDF) nanofibers is reported. The PES exhibits a high sensitivity to pressing and bending, and there is a stable correlation between bending angle and piezoelectric voltage. The sensitivity can be adjusted by changing the doping concentration of GR. Also, when the PES contacts a source of heat, a pyroelectric signal can be acquired. The positive correlation between temperature and signal can be used to avoid burns. The integrated sensing system based on multiple PESs can accurately recognize the action of each finger in real time, which can be effectively applied in sign language translation. PES-based motion-tracking applications have been effectively used, especially in human–computer interaction, such as gesture control, rehabilitation training, and auxiliary communication.

## Introduction

Sign language, as a communication method that works based on gestures, plays an important role for people who are hearing-impaired or unable to speak. With the development of society, the requirement to facilitate the communication with people with hearing or speaking impairments is increasing. Real-time translation systems based on gestures are gradually showing rich application prospects in sign language learning and daily communication. A recently reported yarn-based stretchable sensor can already translate a large number of gestures into speech signals [1–6]. However, traditional real-time sign language translation systems are limited by their bulky design and complex configurations, and always have limitations in terms of portability, comfort and cost [7–16]. A convenient and sensitive sign language translation system is urgently required to meet the needs of daily work and life.

Nowadays, pressure and bending angle sensors are mainly based on signals caused by a changing force [17–25]. Plenty of measurement methods, using different materials and different principles, have been proposed in recent years [26]. Although these sensors can detect various interactions between humans and machines, the indispensability of external power sources greatly narrow their application scopes [27–35]. Piezoelectric sensors generate self-responsive electrical signals based on external mechanical forces. As a self-powered sensing system, piezoelectric sensors show potential in wearable sensing applications [19,36–41]. However, traditional piezoelectric sensor devices such as piezoelectric ceramics have disadvantages in detecting bending, and their detection stability and measurement range need to be improved [18,42–48]. In the era of smart sensing, there is an increasing need for self-powered pressure and bending sensing systems [49–64].

In this study, we propose a flexible self-powered piezoelectric sensor (PES) based on graphene (GR)-doped PVDF nanofibers. The fiber properties after electrospinning were measured, and a potential application of the PES in the translation of sign language was successfully demonstrated. The designed PES shows a high sensitivity regarding both pressure and bending. In particular, a stable angle mapping under bending could be obtained. The amount of GR doping has an impact on sensitivity. In addition, the sensor will generate a large pyroelectric signal when it is touched with a hot object, which can be used to prevent burns of the hands. When the PES is integrated in a measuring circuit, it can accurately perceive the movement state of fingers in real time and output standardized sign language content. This work provides a novel solution for a portable and intelligent sign language translation system, which is considered to be an extremely valuable application to meet the requirements of future intelligent sensing.

## Results and Discussion

**Figure 1:**
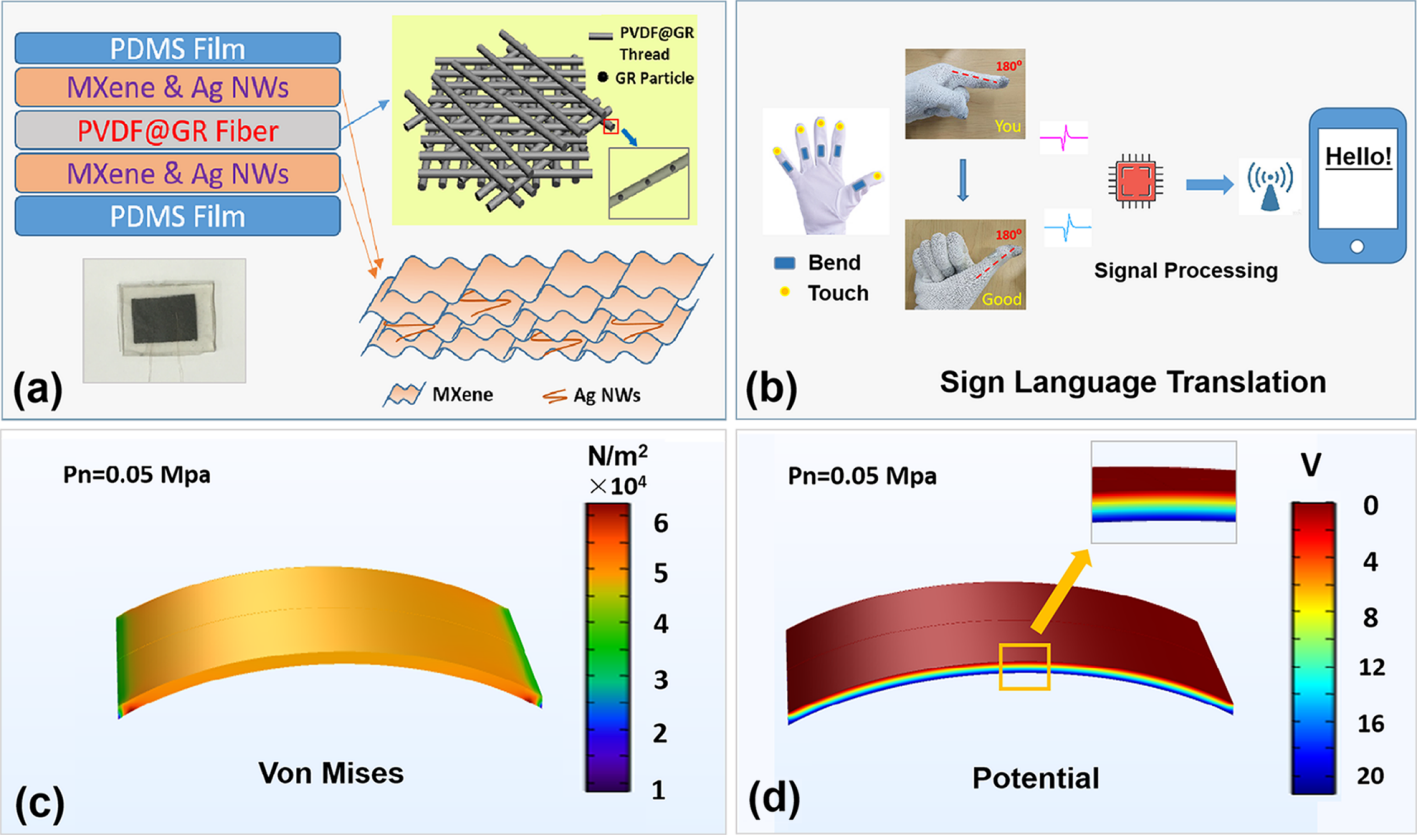
(a) Schematic diagram of a self-powered PES based on GR-doped PVDF. (b) Schematic diagram of an efficient sign language translation system using self-powered PESs. (c) Calculated stress in the PES during bending. (d) Potential distribution in the PES under bending.

The structural design of the self-powered PES based on GR-doped PVDF nanofibers is shown in Figure 1a. The cross section of the self-powered PES shows three parts, namely the GR-doped PVDF piezoelectric layer in the center, the electrode layer of Ti_3_C_2_ MXene and Ag NWs on both sides, and the PDMS protective layer on the outermost sides. Each PVDF fiber contains GR doping. Figure 1b shows a schematic diagram of a sign language translation system based on a self-powered PES. Sensors are separately integrated into the gloves at the joints of the fingers and the fingertips. When a hand gesture such as “Hello” is made, the signal acquisition and processing module will process the recorded sensor signal. The corresponding sign language content is wirelessly transmitted to a screen for convenient recognition. To further study the performance of this piezoelectric material, a bending-induced piezoelectric model was constructed, and the calculated stress and potential distribution were obtained through finite element analysis, as shown in Figure 1c and Figure 1d. Figure 1c shows the stress distribution under bending. It can be clearly found that the stress is mainly concentrated in the bent part of the material, and the potential is generated on the opposite side of the device.

**Figure 2:**
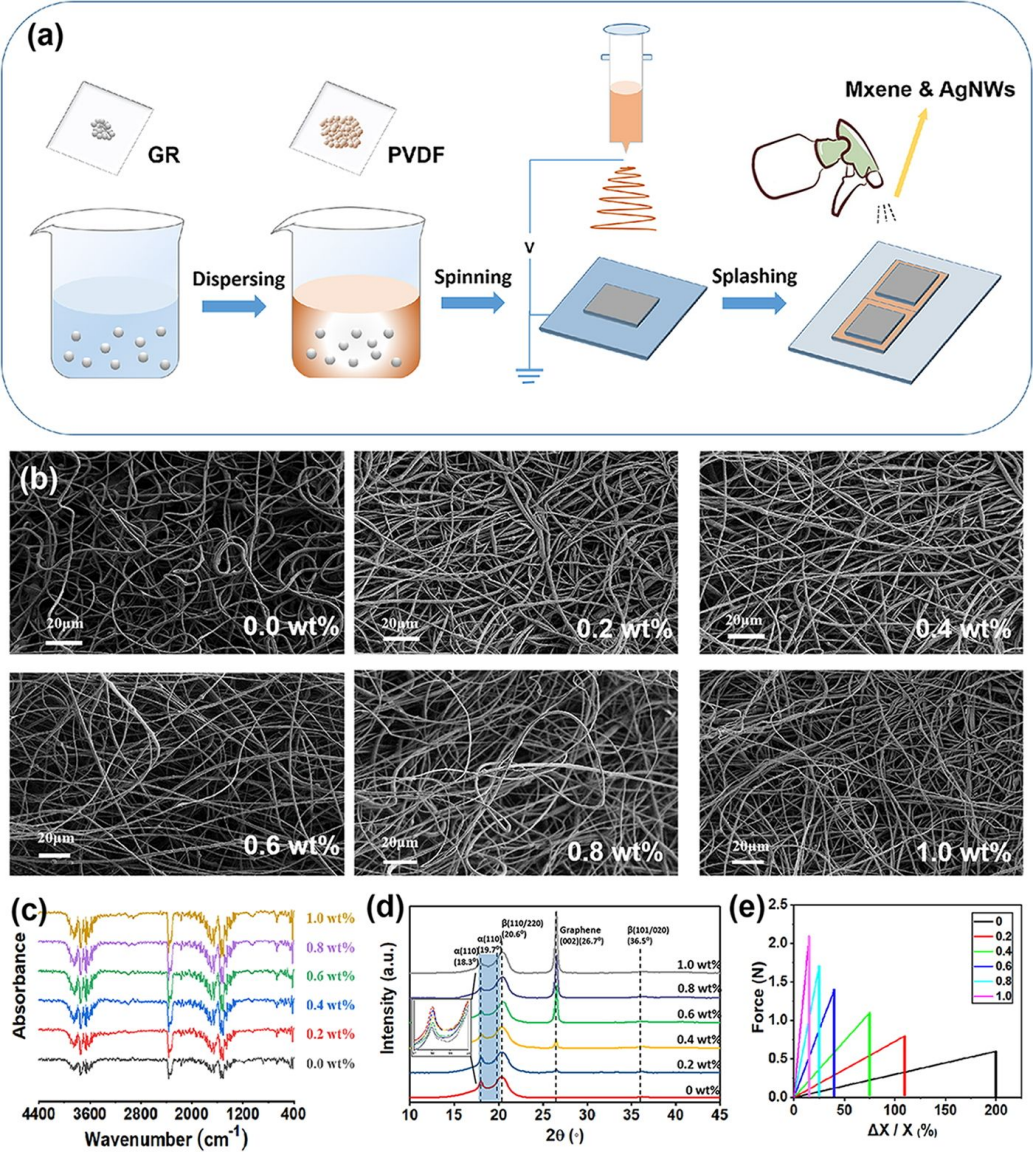
(a) Preparation of the self-powered GR-doped PVDF PES. (b) SEM images of PVDF fibers with different GR doping concentrations. (c) FTIR spectra of the PVDF fibers. (d) XRD patterns of the PVDF fibers. (e) Stress–strain curves of the PVDF fibers.

Electrospinning is used to manufacture GR-doped PVDF fibers. The overall process is shown in Figure 2a. Firstly, GR is dispersed in dimethylformamide (DMF). After ultrasonic treatment, PVDF powder is added under stirring to yield the spinning solution for electrospinning. After preparation of the fibers, an aqueous solution of Ti_3_C_2_ MXene and Ag NWs is sprayed on both sides of the material and then dried. Finally, the nanowire membrane is covered on both sides with PDMS to obtain the piezoelectric sensor. Ti_3_C_2_ MXene and Ag NWs maintain the good conductivity of the electrode and avoid possible short-circuit problems occurring after magnetron sputtering. Also, a stable flexibility of the structure is maintained. GR is added with six different mass fractions, that is, 0, 0.2, 0.4, 0.6, 0.8, and 1.0 wt %. Figure 2b shows SEM images after doping with different concentrations. It can be found that all spinning solutions yield a uniform fiber film without GR agglomeration after electrospinning. Figure 2c shows the FTIR spectra of samples with different doping concentrations. Further, XRD was used to characterize the material (Figure 2d). It was found that as the GR concentration increases, the fraction of the β-phase of PVDF also increases, which is considered to have a positive effect on the piezoelectricity of PVDF. The influence of the GR concentration on the tensile strength of the fibers is shown in Figure 2e. As the concentration increases, the tensile strength of the material increases.

**Figure 3:**
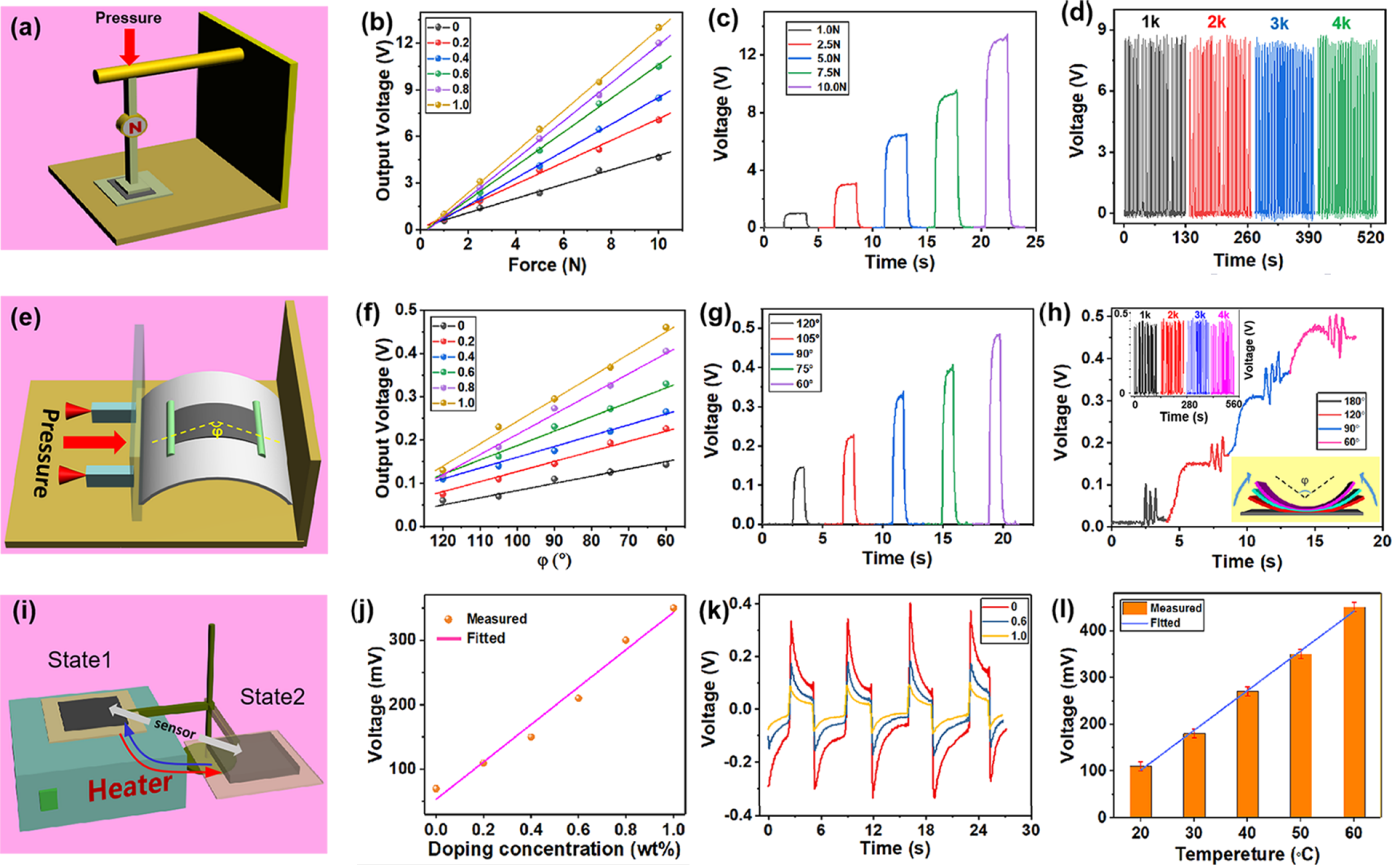
(a) Schematic diagram of the PES under external pressure. (b) Output voltage as a function of the applied pressure for different doping concentrations. (c) Waveforms corresponding to different pressures (1 wt % GR). (d) Waveforms after cyclic pressing. (e) Schematic diagram of the PES under bending. (f) Output voltage as a function of the bending angle for different doping concentrations. (g) Waveforms corresponding to different bending angles (1 wt % GR). (h) Waveforms when the PES is slightly shaken under increasing bending angles. (i) Schematic diagram of the PES contacting a heat source. (j) Output voltage during contact with the heat source for different doping concentrations. (k) Waveforms during contact with the heat source at different concentrations. (l) Output voltage at different temperatures (1 wt % GR).

The output of the self-powered PES was measured with a series of experiments. Figure 3a,b shows that when the pressure gradually increases, the generated piezoelectric voltage increases. The voltage also increases with increasing GR doping concentration, which is consistent with previous characterization results [24]. Figure 3c shows the measured waveform of the device with 1 wt % GR under pressure. After repeated pressing for thousands of times the output of the sensor shows no visible attenuation (Figure 3d). The output voltage increases with stronger bending, that is, smaller bending angles φ (Figure 3e). The effective working range of the sensor under bending is 120° to 60°, and its angular resolution can reach 0.006 V/° at 1 wt % GR. Figure 3g shows the measured waveform of a device with 1 wt % GR under different bending angles. The voltage in Figure 3h was measured while the sensor was gradually bent and shaken at different angles. The output voltage is stable and there is a good mapping relationship with the bending angle. In the upper left corner of Figure 3h, the voltage under repeated bending is shown. The output of the PES shows no obvious attenuation after a large number of bending tests. The PES was attached alternatively to a heater and a cooling fin to measure its pyroelectric voltage (Figure 3i). The heater was kept at a constant temperature of 50 °C. Figure 3j displays that the voltage increases with increasing doping concentration. The pyroelectric voltage of the PES as a function of the doping concentration is plotted in Figure 3k, showing that the measured waveform is stable and repeatable. When the temperature of the heater is changed, the voltage rises as the temperature is increased (Figure 3j).

**Figure 4:**
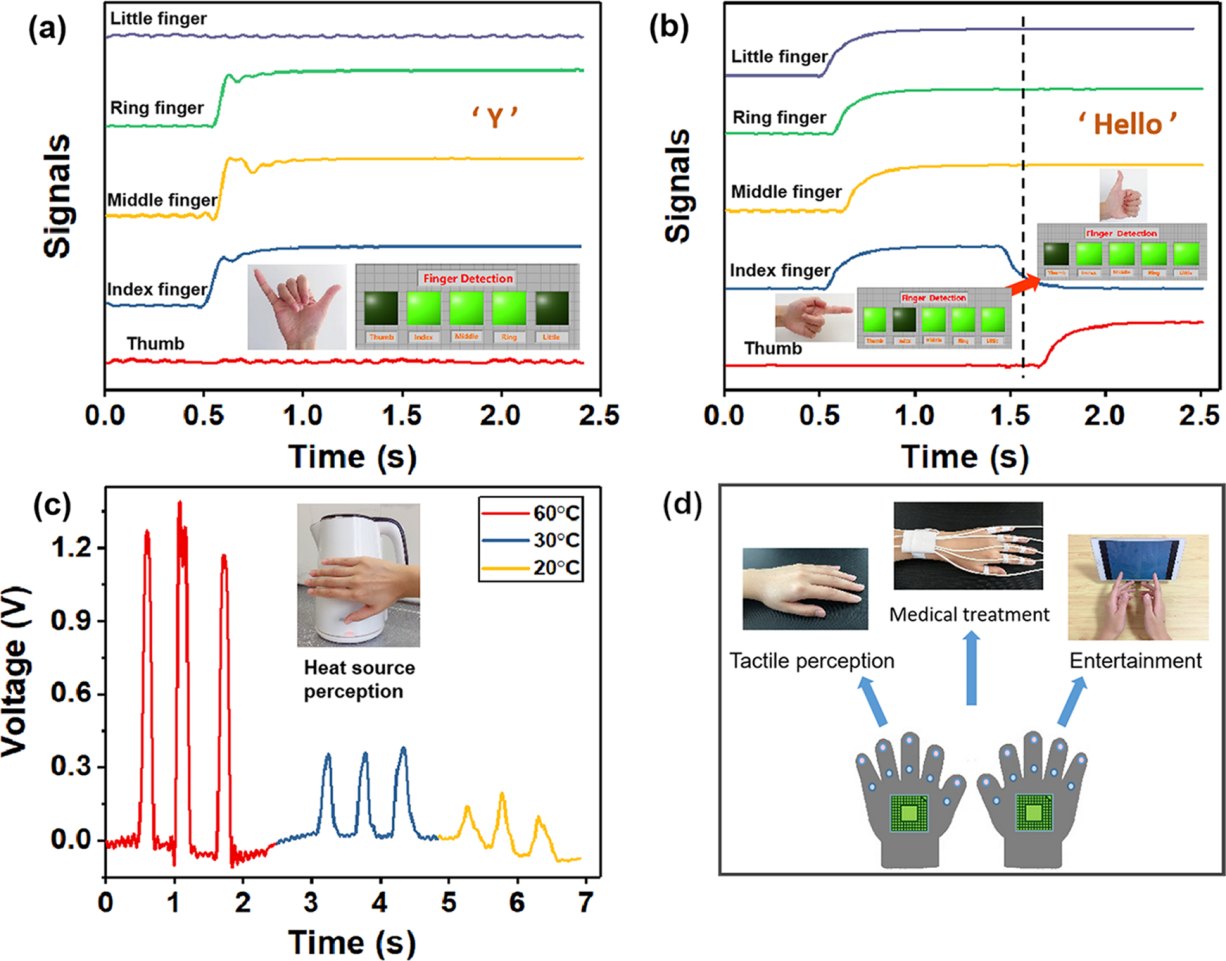
(a) Output signal when a sign language “Y” is shown. (b) Output signal when a sign language “Hello” is shown. (c) Output signal when PES touches a heat source. (d) Potential applications of smart gloves containing PESs.

The PES has great application potential in self-powered motion tracking. To examine its function in sign language translation, PESs were attached to all fingers and the wrists to realize motion tracking through the integrated sensor system. When the hand shows a sign language “Y”, as shown in Figure 4a, the bent fingers produce an output signal and the system recognizes the corresponding sign. When the sign language “Hello” is shown, the corresponding finger action can be well recognized as the fingers move (Figure 4b). The output signal yields an accurate mapping relationship to the hand motion. The output voltage of the PES when it touches a heat source is plotted in Figure 4c. A temperature of 60 °C induces a high output voltage, which can be used to avoid burns of the hands. If PESs made of GR-doped PVDF are integrated into a smart glove, the self-powered sensor system can be used for other applications, such as tactile perception, medical rehabilitation, and gesture games (Figure 4d).

## Conclusion

A self-powered PES based on GR-doped PVDF has been fabricated. Its sensitivity to pressure and bending was examined, especially regarding the reliable measurment of the bending angle. Also, the effect of different doping concentrations on the sensitivity is shown. The pyroelectric voltage can be used to prevent burns. The PES-based sign language recognition system has a good recognition effect for different actions. Our work has paved the way for wearable motion-tracking systems based on piezoelectric sensors, which are of great value in fields such as human–machine interaction, medical rehabilitation, and virtual reality.

## Experimental

**Chemicals:** PVDF (*M*_w_ = 2.75 × 10^5^ g/mol) powder was purchased from SOLEF (USA). *N*,*N*-Dimethylformamide (DMF) was provided by China National Pharmaceutical Group. Graphene powder was purchased from Suzhou Carbon Fung Graphene Technology Company. Ag NWs (60 nm in diameter) were purchased from Lit Nanotech. Ti_3_C_2_ MXene was purchased from Beijing Beike New Material Technology Company. Polydimethylsiloxane (PDMS) and curing agent were purchased from Shenzhen Oss Corporation.

**Electrospinning process:** First, GR is added to DMF and ultrasonically dispersed for 10 min. The dispersion is then heated to 60 °C. PVDF powder (18 wt %) is added under continuous heating and stirring for 6 h to obtain the spinning solution. The prepared solution is filled into a 10 mL syringe with an 18 gauge needle, the rotation speed of the plate is 50 min^−1^, the distance between needle and plate is 15 cm, the injection speed is 0.5 mL/h, the applied voltage is 18 kV, and each sample is electrospun for 5 h.

**Preparation of PES sensor:** Ti_3_C_2_ powder (0.1 g) and Ag NWs (0.05 g) are ultrasonically dispersed in deionized water (10 mL) for 1 h to obtain the spray solution. The liquid is transferred into a spray can and sprayed evenly on the surface of the fiber membrane. Then, the fiber membrane is dried at 60 °C for 5 min. After attaching copper wires on both sides, the membrane is encapsulated with PDMS.

**Thermoelectric test process:** The PES unit is attached to a thin polyethylene plate and connected to the analogue signal test system via the copper wire electrodes. The surface of the plate heater is kept at a temperature of 50 °C. The polyethylene plate is fixed to a vertical rod on one side and can be rotated laterally. The PES is moved between the heating stage and the suspended cooling end in a cycle of approximately 7 s, and the output of the analogue signal is recorded.

**Sign language translation system:** The PESs were attached to five finger bending joints of a glove with double-sided tape. The thin copper wires were connected to the input terminals of the analogue measurement channels of a National Instruments BNC-2111 connector block. Voltage and current were measured with a Keithley 6514 electrometer (200 TΩ input impedance). The analogue signal of each channel is collected in real time. When the voltage exceeds the threshold, the corresponding Boolean indicator lights up, otherwise the indicator light goes out. Thus, the indicator lights can represent sign language gestures.
